# Guideline Development for Technological Interventions for Children and Young People to Self-Manage Attention Deficit Hyperactivity Disorder: Realist Evaluation

**DOI:** 10.2196/12831

**Published:** 2019-04-03

**Authors:** Lauren Powell, Jack Parker, Val Harpin, Susan Mawson

**Affiliations:** 1 University of Sheffield Sheffield United Kingdom; 2 Sheffield Children's NHS Foundation Trust Sheffield United Kingdom

**Keywords:** attention deficit disorder with hyperactivity, technology

## Abstract

**Background:**

Attention deficit hyperactivity disorder (ADHD) is a complex neurodevelopmental disorder characterized by inattention, hyperactivity, and impulsivity. ADHD can affect the individual, the individual’s family, and the community. ADHD is managed using pharmacological and nonpharmacological treatments, which principally involves others helping children and young people (CAYP) manage their ADHD rather than learning self-management strategies themselves. Over recent years, technological developments have meant that technology has been harnessed to create interventions to facilitate the self-management of ADHD in CAYP. Despite a clear potential to improve the effectiveness and personalization of interventions, there are currently no guidelines based on existing evidence or theories to underpin the development of technologies that aim to help CAYP self-manage their ADHD.

**Objective:**

The aim of this study was to create evidence-based guidelines with key stakeholders who will provide recommendations for the future development of technological interventions, which aim to specifically facilitate the self-management of ADHD.

**Methods:**

A realist evaluation (RE) approach was adopted over 5 phases. Phase 1 involved identifying propositions (or hypotheses) outlining what could work for such an intervention. Phase 2 involved the identification of existing middle-range theories of behavior change to underpin the propositions. Phase 3 involved the identification and development of context mechanism outcome configurations (CMOCs), which essentially state which elements of the intervention could be affected by which contexts and what the outcome of these could be. Phase 4 involved the validation and refinement of the propositions from phase 1 via interviews with key stakeholders (CAYP with ADHD, their parents and specialist clinicians). Phase 5 involved using information gathered during phases 1 to 4 to develop the guidelines.

**Results:**

A total of 6 specialist clinicians, 8 parents, and 7 CAYP were recruited to this study. Overall, 7 key themes were identified: (1) positive rewarding feedback, (2) downloadable gaming resources, (3) personalizable and adaptable components, (4) psychoeducation component, (5) integration of self-management strategies, (6) goal setting, and (7) context (environmental and personal). The identified mechanisms interacted with the variable contexts in which a complex technological intervention of this nature could be delivered.

**Conclusions:**

Complex intervention development for complex populations such as CAYP with ADHD should adopt methods such as RE, to account for the context it is delivered in, and co-design, which involves developing the intervention in partnership with key stakeholders to increase the likelihood that the intervention will succeed. The development of the guidelines outlined in this paper could be used for the future development of technologies that aim to facilitate self-management in CAYP with ADHD.

## Introduction

### Attention Deficit Hyperactivity Disorder, Prevalence, and Management

Attention deficit hyperactivity disorder (ADHD) is a highly comorbid [1] neurodevelopmental disorder, defined by 3 core symptoms: inattention, hyperactivity, and impulsivity. It has a worldwide prevalence of 3% to 5% in school-age children [2] and children and young people (CAYP) are most likely to be diagnosed with ADHD in the United Kingdom when they are at primary school [3]. This amounts to approximately 26 million children and adolescents, and this figure is rising globally [4]. Over the last 30 years, the number of people treated for ADHD in the United Kingdom has risen from 0.5 per 1000 to 30 per 1000 [3], and the annual health care costs for young people with ADHD in the United Kingdom are estimated at £670 million. CAYP with ADHD experience a number of ADHD-related difficulties including poor academic attainment, poor social relationships, increased likelihood of being suspended or expelled from school, and leaving school earlier than their peers [5]. In addition, genetic and contextual circumstances can also have an impact on the prevalence of the condition. ADHD is highly heritable [6], and those who are more socially disadvantaged are more likely to be diagnosed with ADHD [7,8]. Moreover, ADHD often continues to affect individuals into adult life [1,9].

ADHD management includes a combination of behavioral and pharmacological interventions [1]. There is strong evidence that pharmacological treatment and nonpharmacological interventions such as psychoeducation programs, behavioral interventions, and cognitive behavioral therapy have a major beneficial effect on the core symptoms of ADHD in approximately 80% of cases, at least in the short term [1,10]. ADHD can affect every aspect of an individual’s life, and support from professionals and family members is limited. There is some evidence of short-term efficacy in managing the core ADHD symptoms, conduct disorders, social skills, self-efficacy, and emotional outcomes. However, CAYP often rely on clinicians and parents to manage the condition of members of the target population, and young people are often unwilling to engage in treatment [11], which limits ADHD self-management into adulthood [12]. Therefore, to attempt the prevention of the individuals falling into crisis when they reach adulthood, it is essential that the members of target population should learn how to self-manage their condition through co-designed interventions [13,14]. This includes exploring contemporary, innovative, and interactive methods of engaging CAYP with ADHD, such as the use of technology may improve their motivation and adherence to treatment. However, methodological limitations make it difficult to draw definitive conclusions from clinical trials [15].

### Self-Management in Children and Young People With Attention Deficit Hyperactivity Disorder and Behavior Change Theories

People with long-term conditions (including ADHD) spend around 1% of their time interacting with a clinician, leaving 99% of their lives managing their condition themselves [16]. However, to self-manage a condition, behavior change is required. A number of theories have attempted to breakdown aspects of behavior that require change. For example, the Chronic Care Model (CCM) [17] identifies 6 elements [18] that are important factors for successful chronic care and prevention management that have previously been applied to the care of CAYP with ADHD [19,20]. These include and are not limited to the following:

1. The promotion of safe quality care; any self-management intervention for CAYP with ADHD will need to adhere to quality standards to ensure the content is reliable and appropriate.
2. Support should be based on evidence and what the patient’s needs and preferences are; if the intervention does not adhere to what the patient wants or needs, the patient may be less likely to engage with it.
3. Self-management support should be provided to help patients manage their health and care; CAYP with ADHD should self-manage their condition to decrease the likelihood of them falling into crisis later in life.
4. Community resources should be available to improve access; resources should be available to facilitate and support the self-management of ADHD in CAYP.

Similarly, the Behavior Change Wheel (BCW) [21] provides a framework specifically for behavior change interventions and involves the *Capability Opportunity Motivation-Behavior* (COM-B) model, which refers to the interactions among “Capability,” “Opportunity,” “Motivation,” and “Behavior.” Capability refers to the psychological and physical ability to engage with an activity, opportunity refers to factors outside of the individual to ensure behavior change is possible, and motivation refers to brain processes that “energize and direct behavior.” The COM-B model provides a useful framework of elements that influence behavior change and can indeed be applied to self-management. For example, to self-manage a condition, the individuals’ behavior will need to change. To do this, they should be motivated and have the capability to change their behavior and be in the correct environment for the change to occur.

Furthermore, the Health Foundation states that people with long-term conditions need to have the knowledge, skills, and confidence to manage their condition “effectively in the context of...everyday life” [22]. These underlying principles of self-management and the principles from the CCM and the BCW are important for all long-term condition self-management, including ADHD in CAYP.

### Technology Interventions for Attention Deficit Hyperactivity Disorder Self-Management in Children and Young People

Technology has been shown to have a large potential to improve the effectiveness and personalization of mental health interventions [15]. A number of attempts have been made to harness the technology to engage CAYP in self-managing their ADHD [23-41]. Examples include a handheld organizational device [37], computer games [25,27,34,39,41], programs [29,30,40], an augmented reality serious game [23], mobile apps to improve reading speed [33], executive functioning [35], and healthy sleep habits [38].

The results of these interventions have found an increased ability to remain on task at school [32], improved organizational skills [37], ADHD symptoms, and sleep [38]. It must also be noted that although a number of these studies have found positive results, it is unclear if these effects are maintained over a longer time period [24,31,33-36].

However, not all of these studies show positive or significant results. ADHD is a highly complex comorbid condition and it is therefore difficult to control for contextual differences using randomized controlled trial (RCT) methodologies. It is also possible that the uptake of each intervention among participants may vary [15]. Others may use the interventions in different contexts to one another with variable distractions [42,43].

### Evaluating Complex Conditions

It is now understood that the steps taken for increasing evidence in complex conditions is no longer linear, and the updated Medical Research Council (MRC) Framework (2008) [44] places greater attention to the context in which interventions take place.

**Figure 1:**
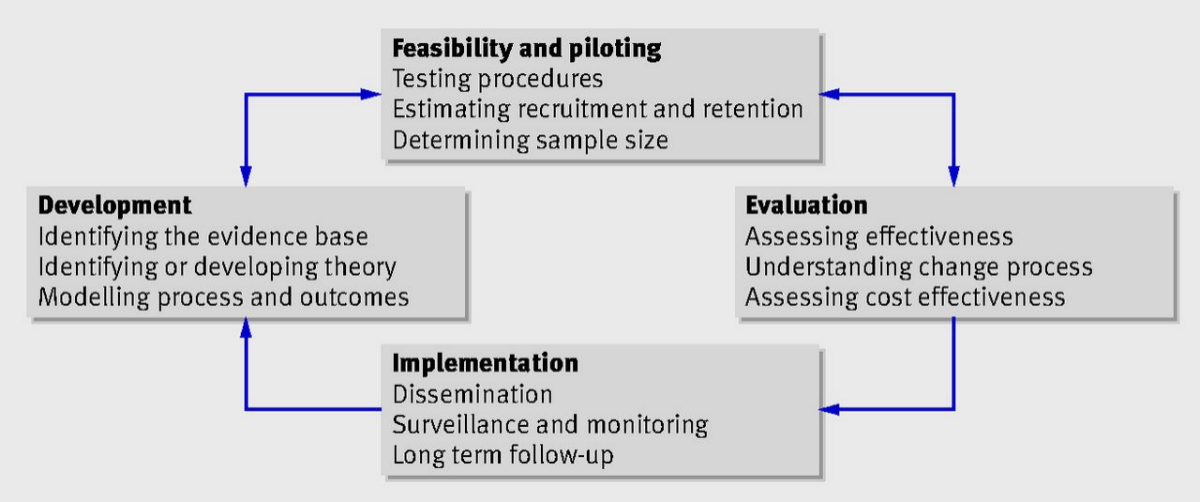
Outlining the Medical Research Council model of complex intervention development.

Figure 1 shows the outlining the Medical Research Council model of complex intervention development [44].

A total of 3 key components for the development of these complex interventions are outlined below:

1. Interventions should be clearly underpinned by existing theories. Theories that are based on existing knowledge can offer a clear way to underpin a rationale, which can assist with communication with stakeholders [45,46].
2. Interventions should be developed in partnership with key stakeholders [46-48].
3. Intervention developers should account for the context by which the intervention is developed by identifying what works for whom and under what circumstances. This means the intervention is more likely to be a success [42,43,49].

Other evaluation study designs such as RCTs and quasi-experimental studies only answer the question “What works?” and do no capture the complexity of complex conditions and interventions or the characteristics of the context in which the intervention is delivered [50]. This is important as the context, content, and outcomes of a complex intervention can involve a high degree of variance [50]. Therefore, if technological interventions are designed to be used with complex conditions such as ADHD in various contexts, it is imperative they are underpinned by theory and consider the contexts in which the intervention will be delivered [44]. A previous attempt to develop a complex intervention went beyond the question “What works?” and this involved a realist review that explored the question “what works for whom, under which circumstances and respects” [51]. However, to our knowledge, a realist evaluation (RE) has never been used to develop guidelines for the development of interventions *.*

Therefore, this study aimed to utilize an RE methodology [52] and involve key stakeholders (CAYP with ADHD, their parents and or carers, and specialist clinicians) in the development of theory- and evidence-based guidelines. The guidelines developed may help the future development of technological interventions that aim to help primary school–aged CAYP with ADHD self-manage their condition more effectively. Primary school–aged CAYP have been chosen as this is the most common age to be diagnosed with ADHD in the United Kingdom. RE aims to go beyond the “what works” question and answer the question “what works for whom, under which circumstances and respects.” RE also takes into account the complexities of the condition, the intervention, and the context by which it is delivered [52]. The use of underpinning behavior change middle-range theories (MRTs; see Table 1 for definition) will improve the generalizabiliy of the guidelines to more than 1 context. There is a need for these guidelines as existing frameworks are useful in terms of generalization to many conditions whereas CAYP with ADHD have complex needs that need addressing separately to ensure future interventions are suitable for them.

**Table 1:** Definitions of context, mechanism, and outcomes.

Term	Definition
MRT^a^	A theory that can be used to explain specific parts of an intervention is called an MRT. MRTs are identified at the beginning of this process and examined throughout the process and for this study, during data collection.
Context	The environment or “backdrop” of an intervention is called Context. Context can change over time, which could reflect aspects of change while an intervention is implemented [54]. The context may limit or allow the mechanisms.
Mechanism	This refers to aspects (“resources”) that are a result of the intervention and the response to those resources, for example, cognitive, motivational, and emotional [54].
Outcome(s)	Outcomes (intended or unintended) refer to what may happen because of an intervention. For example, variable context may create an unintended outcome, which could be vital to intervention delivery.

^a^MRT: middle-range theories.

## Methods

### Principles of Realist Evaluation

**Figure 2:**
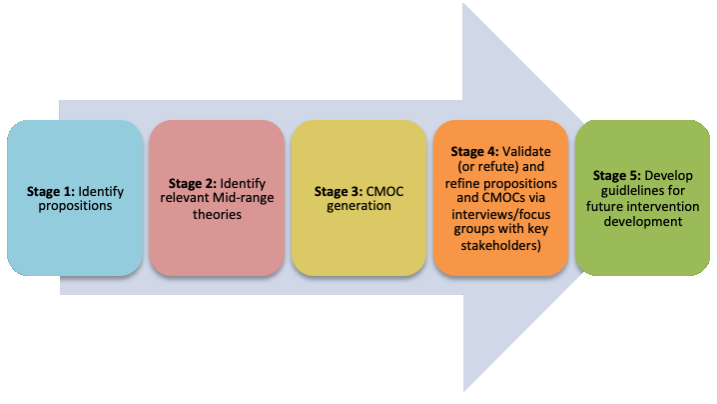
Outlining the process of generating, validating and refining propositions and context mechanism outcome configurations. This process lasted between May and September 2018.

RE has been shown as an effective framework for evaluating complex health interventions [43]. The aim of RE is to explore how a mechanism may cause a different outcome when in different contexts (see Table 1 for definitions) [52]. The process adopted for this study is outlined in Figure 2. The RE approach outlined in this study has been guided by Realist And Meta-narrative Evidence Synthesis: Evolving Standards II reporting standards for RE [53] and has been followed by the process stipulated in Pawson et al’s study, 1997 [52].

### The Five Stages of This Study

The 5 stages of this study are as described in the following sections:

#### Stage 1: Identifying Propositions

Propositions are comparable with that of hypotheses that predict what is believed to occur in a given situation or within research. Developing the propositions for this study involved authors LP and JP exploring theoretical concepts from the literature that derives from behavior change and human-computer interaction theories (see Table 2) that could underpin a technological intervention that aims to help CAYP with ADHD self-manage their condition. Agreement of these concepts was reached through discussion among all the authors. The product of Stage 1 was a list of propositions.

#### Stage 2: Identifying a Theoretical Framework

Using the principles of RE [43], a theoretical framework was formed to underpin the development of the intervention guidelines, that is, concepts within identified theories could underpin specific components (or “mechanisms”) of an intervention. The theoretical framework was based on theories that can be applied to educating CAYP with ADHD and human computer-interaction (see Table 2), and it was constructed by authors LP and JP.

#### Stage 3: Context Mechanism Outcome Configuration Generation

After the propositions (Stage 1) and the theoretical framework (Stage 2) were developed, they were set out as context mechanism outcome configurations (CMOCs) during Stage 3 of this process. Authors LP and JP generated the CMOCs. Table 3 outlines some examples of the CMOCs generated during Stage 3.

**Table 2:** Product of Stage 2: demonstrating how middle-range theories underpin the intervention guidelines.

Middle-range theories	Ingredients and middle-range theory link	How intervention could incorporate the ingredients
CC^a^, OC^b^, OST^c^, SLT^d^, SRT^e^, ED^f^, DDT^g^, ELT^h^, SCT^i^, SDT^j^, OIT^k^, BCW^l^, CCM^m^	Reward (OC, CC, DD, SDT, BCW)	Immediate rewards for all correct responses to engage and motivate the user.
	Stimulation (OST, ED)	User can move on to different available sections of the intervention and previous work will be saved to return to later. User has the choice to carry out intervention activities electronically or on paper.
	Sequential learning (ED)	All “sections” of intervention to not be available at once (preventing overstimulation). Different sections become “unlocked” once other sections are completed.
	Self-efficacy (SLT and SCT)	Intervention will provide the users with the opportunity to self-evaluate their performance, by receiving feedback from the intervention (eg, stars and coins) and from others (verbal persuasion or encouragement).
	Learning (ELT)	Paper-based activities will be available for those with limited access to a device (eg, sharing with siblings or limited device access at bedtime) and/or internet.
	Independent practice (SLT)	Used in the absence of a clinician.
	Social regulation (SRT and CCM)	Section that teaches user techniques to self-manage ADHD^n^, for example, anger management.
	Social Learning (SLT)	Intervention should provide scenarios of social situations where the user can make appropriate decisions (reinforced with immediate rewards).
	Social cognition (SCT)	Setting short-term, meaningful, and relevant goals for the users to motivate them to engage with the intervention.
CD^o^, UID^p^, and CCM	Stakeholder involvement in design (CD, UID, and CCM)	Stakeholders should be involved in the design and development of the intervention to increase intervention success.
CC, OC, OST, SLT, SRT, ED, DDT, and ELT.	Self-monitoring	Users monitor their performance independently.
	Reinforcement	Intervention should provide positive feedback where applicable and they can share this with others.
	Self-management	Intervention should give the users opportunities to problem solve, make decisions, and take action in real life scenarios based on what they have learned.
	ADHD Knowledge and understanding	Intervention should provide the users with accessible information to help them better understand ADHD so they can more optimally self-manage it.

^a^CC: Classical Conditioning [55].

^b^OC: Operant Conditioning [56].

^c^OST: Optimal Stimulation Theory [57].

^d^SLT: Social Learning Theory [58].

^e^SRT: Social Regulation Theory [59].

^f^ED: Executive Dysfunction [60].

^g^DDT: Dynamic Developmental Theory [61].

^h^ELT: Experiential Learning Theory [62].

^i^SCT: Social Cognitive Theory [63].

^j^SDT: Self Determination Theory [64].

^k^OIT: Organismic Integration Theory.

^l^BCW: Behavior Change Wheel [21].

^m^CCM: Chronic Care Model [17].

^n^ADHD: attention deficit hyperactivity disorder.

^o^CD: Co-design [13,14].

^p^UID: user interface design.

**Table 3:** Product of Stage 3: context mechanism outcome configuration examples.

CMOCs^a^	Plausible mechanism: “What”	Contexts: “for whom” and “in what circumstances”	Possible outcomes
CMOC 1	Receiving feedback from the intervention might improve the users’ confidence by confirming performance.	Internet and intervention accessible at home, used independently of clinician.	Development of self-efficacy
CMOC 2	Positive reinforcement (reward) may motivate the user to use the intervention.	Intervention should give positive rewarding feedback to the user.	Increased understanding of condition and self-management

^a^CMOC: context mechanism outcome configuration.

#### Stage 4: Validation and Refinement of New and Existing Context Mechanism Outcome Configurations

CMOCs were then validated and refined by conducting interviews with CAYP with ADHD, their parents/carers, and specialist clinicians. Author LP conducted the interviews and they were conducted at the participant’s convenience. Clinician interviews were undertaken at the clinicians’ workplace and young persons’ and parents’ interviews took place in their homes.

##### Participants

Participants were recruited to adhere to the sampling frame below.

- CAYP with ADHD and their parents/carers
  - Males and females
  - CAYP with autism spectrum disorder (ASD) and without ASD
  - Families who live in the 10% of most and least deprived areas of the United Kingdom [65]
  - CAYP with ADHD aged 8 to 11 years
- Clinicians
  - A sample that includes ADHD specialist nurses, a pediatrician, and a psychiatrist.
  - Clinicians who work at Child and Adolescent Mental Health services and pediatric neurodisability services.

##### Recruitment

CAYP with ADHD and parents/carers were recruited via a database held by the research team. Clinicians were recruited via the National Health Service (NHS) in the South Yorkshire region. Participants were recruited until data saturation was achieved [66]. The eligibility criteria for CAYP with ADHD were (1) aged 8 to 11 years and (2) diagnosed with ADHD. Parents/carer (1) must have been a parent/carer of a young person with a confirmed ADHD diagnoses and (2) must have been able to provide details of the ADHD medication the young person was prescribed. Clinicians had to be employed by a service that treats CAYP with ADHD and has experience of working with this population.

##### Procedure

Semistructured interviews focused on initial propositions that were tested and refined. CAYP with ADHD, their parents/carers, and clinicians provided interview data to test the propositions. The study received ethical approval from the University of Sheffield’s School of Health and Related Research Ethics Committee (Ref: 021203) and received NHS Health Research Authority and Research and Development local approval. Interviews took place in the CAYP/parents/carers’ homes and clinicians’ workplaces. All participants provided written informed consent or assent (CAYP only).

Participants were asked (age appropriate) questions that were derived from the propositions. Questions included the following:

- What type of feedback do you think your child would like and why? (parent/carer)
- What do you think the role of friends and family could be for supporting CAYP with ADHD with a technological intervention? (clinicians/parent/carer)
- If you play a computer game, do you like to collect things like coins, stars, points? (CAYP with ADHD)

Parents/carers provided ADHD medication details for their child (where applicable) and completed a Swanson Nolan and Pelham IV questionnaire to provide a measure of their child’s current ADHD symptoms.

##### Data Analysis

Analysis focused on refining and generating new CMOCs. Principles of thematic and framework analysis were adopted [67,68]. Guidelines were identified on the basis of existing CMOCs (framework analysis approach), and when data did not fit with existing CMOCs, new CMOCs were generated (thematic analysis approach) [43].

#### Stage 5: Development of Guidelines

This was based on the refined and newly generated CMOCs from Stage 4. The final guidelines aim to provide a set of recommendations for designing a complex technological intervention that aims to help CAYP with ADHD self-manage their condition. The guidelines also provide advice regarding the environment in which the intervention should be delivered. The CMOCs refined during Stage 4 were used to form the content of the guidelines. The guidelines can be found in Multimedia Appendix 1. Author LP initially put the guidelines together and then discussed the guidelines with the rest of the research team (authors JP, VH, and SM) and refined them accordingly.

## Results

### Participant Characteristics

A total of 21 participants (7 CAYP, 8 parents, and 6 clinicians) were recruited from July 2018 to October 2018. Participant demographic information is included in Table 4 (CAYP), Table 5 (parents), and Table 6 (clinicians). All parents were able to provide information regarding their child’s ADHD medication. All interviews were transcribed verbatim. During analysis, agreement between the 2 primary coders was high.

### Initial Propositions (Stage 1)

Overall, 9 propositions were identified by author LP and checked for accuracy by author JP. They were then tested against the interview data and refined:

1. If the user receives feedback from the intervention, then the user’s confidence may be improved.
2. If the user can access downloadable resources from the intervention, then the user may generate a deeper understanding of the concepts covered within the intervention.
3. If the users can choose personalizable characters and a variety of modules within the intervention, then this may enable them to maintain stimulation to carry out the task.
4. If the users receive positive reinforcement (reward) from the intervention, then this may motivate them to use the intervention.
5. If the users engage with social scenarios within the intervention, then they may make more appropriate social decisions in the future, which may help enhance social relationships.
6. If the users engage with the intervention, then they may gain a better understanding of their ADHD.
7. If the users engage with the intervention, then improved self-management of their ADHD may improve relationships with friends and family.
8. If the user gains encouragement from friends/relatives to use the intervention, then this could reinforce the user’s engagement with the intervention.
9. If short-term meaningful goals are set for the users via the intervention, then this could encourage them to engage with the intervention.

**Table 4:** Demographic information of children and young people with attention deficit hyperactivity disorder.

Study ID	Gender	Age (years)	Other diagnosis	ADHD^a^ medication	Medicated during interview?	SDI^b^	Inattention SNAP^c^ Score	Hyperactivity or Impulsivity SNAP Score	Connors Index	Combined SNAP Score^d^
YP1	Female	11	ASD^e^	Concerta	Yes	820	2	1.66	1.8	1.82
YP2	Male	9	N/A	N/A	No	13513	2.55	2.89	2.5	2.65
YP3	Female	8	ASD	Usually 27 mg Delmosart	No	17403	3	3	2.7	2.9
YP4	Male	10	N/A	Delmosart 36 mg	Yes	23954	1.78	2	2.6	2.13
YP5	Male	11	N/A	Delmosart 36 mg+27 mg	Yes	4913	1.56^f^	2.22	2.4	2.06
YP6	Male	9	N/A	Delmosart 36 mg	Yes	1318	1.78	2.56	2.2	2.18
YP7	Male	8	Attachment disorder	Elvanse, 40 mg	Yes	32596	1.67	1.88	1.9	1.82

^a^ADHD: attention deficit hyperactivity disorder.

^b^SDI: Social Deprivation Index. 1 is indicative of the most deprived area in the United Kingdom and 32844 is the most affluent area in the United Kingdom.

^c^SNAP: Swanson, Nolan, and Pelham Questionnaire. SNAP Scores: Scores indicative of ADHD are as follows: Inattention: 1.78 and above; Hyperactivity/Impulsivity: 1.44 and above; Connors Index: 1.67 and above; Combined score: 1.63 and above.

^d^Average score across Inattention, Hyperactivity/Impulsivity and Connors Index subsections.

^e^ASD: autism spectrum disorder.

^f^Please note YP6 does not meet the threshold for one SNAP component. They did meet the criteria for all other SNAP domains.

**Table 5:** Demographic information of parents of children and young people with attention deficit hyperactivity disorder.

Participant ID	Comorbid condition of child	Age of child (years)
P1	ASD^a^	11
P2	—^b^	9
P3^c^	ASD	8
P4	—	10
P5	—	11
P6	—	9
P7	Attachment disorder	8
P8^c^	ASD	8

^a^ASD: autism spectrum disorder.

^b^Not applicable.

^c^P3 and P8 are the parents of the same child and were interviewed together.

**Table 6:** Demographic information of clinicians demonstrating 8 months to 18.5 years of experience of working with children and young people with attention deficit hyperactivity disorder with a mean of a total of 6.9 years of experience.

Participant ID	Gender	Job title	Clinical experience with children and young people with attention deficit hyperactivity disorder (years, months)
C1	Male	Registrar psychiatrist	2 years
C2	Female	Consultant pediatrician	4 years 6 months
C3	Female	Nurse clinical specialist	18 years 6 months
C4	Female	Nurse prescriber	8 months
C5	Female	Consultant community pediatrician	10 years
C6	Female	Consultant community pediatrician	6 years

### Testing the Propositions

Overall, 7 themes were identified: (1) positive rewarding feedback, (2) downloadable gaming resources, (3) personalizable and adaptable components, (4) psychoeducation component, (5) integration of self-management strategies, (6) goal setting, and (7) context (personal and environmental). These themes focused on testing the 9 initial propositions.

### Positive Rewarding Feedback (Propositions 1 and 4)

All participants expressed a wish for immediate positive reward when the user may select a correct response. Of the participants, 1 said that when he or she gets a reward, for example, a sticker at school, it makes him or her feel “proud” (YP5). Examples of instant reward could be auditory confirmation of a correct response and collecting items such as coins, stars, diamonds or trophies. The reward (and the intervention itself) should also be visually attractive:

I think that [instant positive reward] will really help his self-confidence.

P4

Another clinician states the following:

I think a lot of the games nowadays build up points and it makes sense...having reward builds up their self-esteem.... And just makes them feel happier.

C4

In addition, all 21 participants suggested that the instant positive reward component would motivate the user to engage with the intervention. They also felt that additional motivation to engage with the intervention could involve personalizing the reward (n=12), that is, the users can choose their rewards (eg, coins, trophies, stars) because of the following reason when referring to CAYP with ADHD:

tend to get bored quite quickly.

C2

A total of 11 participants stated reward could also be given by providing different levels where the use could “level up” or open “new areas” once a previous level is “completed.” Most of the CAYP (n=5) and 2 parents wanted these levels to increase in difficulty:

I like harder and harder cos if you do harder and harder you get better and better at it.

YP7

However, 2 CAYP (YP3 and YP6), 1 parent (P7), and 2 clinicians believed that if levels were too challenging for the users, it could cause frustration and demotivate their engagement with the intervention. Therefore, 1 clinician suggested that there could still be levels and areas to create choice, allowing the users to feel they are progressing, but these levels could have an option to make them easier:

...simplify the challenge so you could make the challenges harder...but there could be a simplify option that the kids could use and so the kids that do get frustrated can simplify it and get it done.

C1

### Downloadable Gaming Resources (Proposition 2)

If applicable, the option of using downloadable resources could be made available for when the intervention may not be accessible, for example, if the child/young person has to share a device with siblings, has limited screen time (eg, before bed time), or is away from home (eg, in the car or on holiday). Participants wanted downloadable resources to have a gaming component, including quizzes, mazes, word searches, crosswords, coloring in, or origami activities (5 CAYP, 3 parents, and 2 clinicians).

Including quizzes cos I like quizzes.

YP1

### Personalizable and Adaptable Components (Proposition 3)

A total of 5 CAYP, 4 parents, and all 6 clinicians requested that the technology should be personalizable and include adaptable avatars, that is, characters they can personalize by changing hair/eye color, gender, clothing, and skin color that can be adapted as and when they wish. Moreover, 4 clinicians believed this was so that the user could “relate” to the intervention and its content:

That it’s [the language] not too clinical and that they can actually relate to it... It’s the relating to it really that’s most important. ...you have to be really careful that its not so generalised that they can’t relate to it.

C3

Moreover, 1 parent (P4), 1 young person (YP4), and 1 clinician (C4) emphasized the importance of having the correct amount of stimulation to ensure the users are not over or under stimulated:

You don’t want to over-stimulate them, but you want them to have that draw, I think its finding the right balance between overload and sort of retaining err concentration.

P4

### Psychoeducation Component (Proposition 6)

A total of 5 CAYP, 5 parents, and all 6 clinicians believed it was an important aim for the users to have a good understanding of their ADHD. It was also considered important by 2 clinicians (C2 and C3) that the positive aspects of ADHD should be highlighted through examples of others who have ADHD and have been successful, such as celebrities, as they believed there was a lot of negativity surrounding the condition. Moreover, 1 clinician believed that it could be “life changing” (C1).

Another clinician stated the following:

Because I want to know about ADHD, what it does and what it effects in your body.

YP5

Moreover, 1 parent stated the following:

Knowledge is power and just giving her the confidence, increased self-esteem.

P3

A clinician stated the following:

I think it could be massive for them across the board it could help them at school, help them learn, help them make friends, help with their relationships with others...

C1

A total of 5 clinicians wanted interventions for CAYP to self-manage their ADHD to be more positive while not “glossing over” some of the difficulties. All 6 clinicians expanded on the above and stated that when CAYP with ADHD act incorrectly or impulsively, they often feel bad about themselves, and having knowledge about their condition could help prevent this. Overall, 3 CAYP, 4 parents, and 4 clinicians emphasized the importance of understanding the users’ ADHD so the users can explain it to their friends:

Cos if I know more I can tell people more about the like what I’ve got [ADHD] so they know what it means.

YP4

### Integration of Self-Management Strategies (Propositions 5 and 7)

Overall, 5 CAYP, 4 parents, and all 6 clinicians believed an intervention should include strategies to help the children self-manage their ADHD, such as anger management strategies. Moreover, 1 participant stated that he counts to 40 for a total of 3 times to calm down (YP7).

Another parent stated the following:

He can learn sort of techniques you know sort of self-management techniques trying to calm himself down.

P4

Another self-management strategy discussed was animated “social scenarios” with alternate endings for the users to choose from to help them understand what acceptable behavior is and is not in social situations. This idea was favored by clinicians (n=5), CAYP with ADHD (n=3), and parents (n=3). Overall, 2 clinicians (C5 and C6) stated this could be beneficial because similar “social stories” are already used with CAYP with ADSD, which is comorbid in many CAYP with ADHD:

She seems to learn a lot through like watching videos... if she wants to know how to do something, she goes on YouTube.

P1

A clinician stated the following:

I really like the idea of scenario-based teaching.

C2

Another clinician said:

[Social scenarios] sound like a similar principle to the social stories we use with the children with autism we see. I think that could be useful as it could help the children to reflect on what they might do in a situation before they are in the heat of the moment.

C5

### Goal Setting (Proposition 9)

Overall, 6 parents liked the idea of goal setting within a technological intervention. They liked the idea of short-term goals because of poor working memory in CAYP with ADHD, which means they may find it challenging to process longer-term goals.

### Context (Propositions 6 and 8)

It was found that the variable context an intervention is delivered in could affect the outcome it may have, and these contexts could be divided into environmental and personal.

**Table 7:** Refined context mechanism outcome configuration examples that support initial propositions.

CMOCs^a^	Plausible mechanism: “What”	Contexts: “for whom” and “in what circumstances”
CMOC 1	Receiving positive rewarding feedback from the intervention might improve the users’ confidence by confirming performance.	Internet and intervention accessible at home and used independently of clinician. Intervention should be colorful and not too text heavy.
CMOC 3	Enabling the user to choose personalizable and adaptable characters of majority and minority groups and a limited number of “modules” will maintain stimulation to carry out the task.	The intervention will give positive and rewarding feedback to the user. Users will also have their own user area so that they can return to previous work and carry on where they left off.

^a^CMOC: context mechanism outcome configuration.

**Table 8:** Additional context mechanism outcome configurations generated from context mechanism outcome configuration validation with key stakeholders.

CMOCs^a^	Plausible mechanism: “What”	Contexts: “for whom” and “in what circumstances”
CMOC 10	Users will have a better understanding of their ADHD so they can explain it to others (friends/family).	The intervention will provide age-appropriate information to improve the users’ knowledge and understanding of their ADHD^b^ and provide suggestions on how to explain their ADHD to others.
CMOC 11	An indication of improvement or progress such as leveling up will motivate adherence.	The intervention will provide the user with varying game levels to keep them engaged and motivated to use the intervention. A “simplify option” will also be available to keep frustration levels down where applicable.

^a^CMOC: context mechanism outcome configuration.

^b^ADHD: attention deficit hyperactivity disorder.

#### Personal Contexts

Overall, 1 parent (P3) and 1 clinician (C5) stated that some CAYP with ADHD also have dyslexia and may struggle to read text; therefore, the background color to any included text should be adaptable. This is because some people with dyslexia find it easier to read text on specific background colors. This could enable the user to access the information more easily. Overall, 1 parent (P1) and all 6 clinicians also believed it is important that the information presented should be developmental and age-appropriate and the language should be suitable to ensure the user can understand the material provided:

A whole variety of those different [background] colours then that would make it much more accessible. It would make it easier for them [CAYP with dyslexia as well as ADHD] to read, it could stop the words and the letters moving, it makes it so they can actually read what’s written rather than it being a sea of text they can’t access. There’s a huge overlap between lots of condition like dyspraxia, dyslexia, ADHD, Autism.

C5

#### Environmental Contexts

Overall, 13 participants (3 CAYP, 4 parents, and 6 clinicians) believed they would be more motivated to engage with a technological intervention if they had encouragement and support from close friends or relatives. Moreover, 1 young person (YP1) stated that her family and her dog could get in the way if she was to use an intervention of this nature, which could affect the effect the outcome intervention has on the user:

I think it will be good for them to do on their own but I think it will be good for other people to know what they have looked at so they can reinforce if they have any questions.

C3

A total of 6 parents believed that supporting their child with an intervention that helps them self-manage their ADHD could help build their relationship with their child.

### Stage 4: Context Mechanism Outcome Configuration Refinement

As a result of validating CMOCs with key stakeholders, existing CMOCs have been refined (see Table 7 for examples) and 2 more CMOCs have been developed (Table 8). All CMOCs can be found in Multimedia Appendix 2.

## Discussion

### Principal Findings

This study aimed to present an RE approach to develop guidelines that may help the future development of technological interventions, which aim to help CAYP with ADHD self-manage their condition more effectively. A total of 7 key themes emerged from the interviews with key stakeholders: (1) positive rewarding feedback, (2) downloadable gaming resources, (3) personalizable and adaptable components, (4) psychoeducation component, (5) integration of self-management strategies, (6) goal setting, and (7) context (environmental and personal). Importance was placed on the variable environmental and personal context in which such an intervention could be delivered; importance was additionally placed on how these contexts could affect the outcomes of the interventions.

### Comparison With Previous Work

#### Positive Rewarding Feedback

All participants identified the need for an instant positive reward within a technological intervention for CAYP with ADHD. This is supported by behavior change MRTs such as classical conditioning, which states that unconscious behavior will change when a stimulus is repeatedly paired with a particular response such as rewards [55]. Similarly, Operant Conditioning is when an individual repeatedly makes an association with a stimulus, such as reward or punishment [56]. These theories explain why the administration of reward can change behavior. Dynamic developmental theory states CAYP with ADHD have a shorter “window” between behavior and a reward response for them to make the association between the behavior and the positive response [61]. This explains why the reward should be immediate. Bandura’s theory of self-efficacy also states that gaining confidence by achieving and accomplishing a task can increase an individual’s self-efficacy. This is referred to as “Mastery Experiences” [69]. The BCW states that the individual needs to be motivated for behavior to change [21], and reward could motivate a child with ADHD to engage with an intervention.

#### Downloadable Gaming Resources

Some participants (5 CAYP, 3 parents, and 2 clinicians) liked the idea of having the option of being able to print off resources that complement the technological intervention in the event that technology is not available (eg, before bedtime and away from the home). This could be important as CAYP with ADHD are overrepresented in socially deprived areas [7,8] and may not have access to technology. It would also provide the user the opportunity to have an experience away from a screen and could help supplement learning by conducting a physical action. The latter claim is supported by John Dewey’s Experiential Learning Theory [62].

#### Personalizable and Adaptable Components

Previous evidence suggests that CAYP with ADHD would like a mobile app to be personalizable [47]. It is well documented that CAYP with ADHD need to be optimally stimulated to maintain engagement with a task [70-72]. As advised by study participants (5 CAYP, 4 parents, and all clinicians), personalizable avatars that are able to be constantly adapted as and when the users would like could provide them with the stimulation and motivation to remain engaged with the intervention. CAYP with ADHD have also been reported to want to adapt avatars so that they can relate to them [47]. A total of 4 clinicians emphasized the importance of the CAYP being able to relate to the intervention. Support for this can come from a “mini theory” Organismic Integration Theory (OIT), derived from self-determination Theory. OIT emphasizes the importance of relatedness to motivate an individual to behave in a certain way [64].

#### Psychoeducation Component

Participants (5 CAYP, 5 parents, and all 6 clinicians) wanted CAYP to know more about their ADHD so that they could self-manage it more effectively and so that the CAYP could explain what ADHD means to their peers. This concurs with existing literature where emphasis has been placed on the value of psychoeducation for CAYP with ADHD and their families, as an expert understanding of their condition could lead to more positive individual choices [49,73]. The Health Foundation reports that educating people about their long-term condition can support self-management [74]. Public Heath England [75], along with the Mental Health Taskforce’s 5 Year Forward View for Mental Health [76], states that early intervention avoids CAYP falling into crisis and expensive longer-term interventions into adulthood. This evidence suggests that psychoeducation for CAYP with ADHD as early as possible is vital to help them understand and self-manage their condition. Despite this favorable evidence base for psychoeducation, CAYP with ADHD often do not have access to appropriate psychoeducation, and their understanding of the condition is frequently poor and likely to lower self-esteem.

#### Integration of Self-Management Strategies

Overall, 5 CAYP, 4 parents, and all 6 clinicians believed the availability of self-management strategies for ADHD could be useful for CAYP with ADHD. Social learning theory states that individuals can learn by imitating others [58]. Animated social scenarios whereby the user can choose alternate endings could enable the user to learn about acceptable behavior in social situations. Bandura’s self-efficacy theory states that “Modeling” can increase self-efficacy [69].

Moreover, 2 clinicians recognized that “social stories” are an effective way to teach CAYP with ASD how to behave appropriately in social situations and are often used in clinical practice [77,78]. Therefore, they believed the proposed animated social scenarios could work well with many CAYP with ADHD, especially those CAYP who have comorbid social skills difficulties.

Furthermore, “interpreting physiological signs” is also a stream of Bandura’s theory of self-efficacy [69]. This could have applied to ADHD in CAYP as if the young people can identify when they are likely to feel angry or frustrated, this could be when they apply some self-management strategies to control their behavior, which could lead to an improvement in their self-efficacy.

As CAYP with ADHD can be impulsive, it was requested that interventions should involve a component to help them when they wish to behave impulsively, for example, when they are angry. Support from this may come from the Social Regulation Theory that states CAYP with ADHD lack self-control, which can affect their working memory [59]. This theme is also supported by the CCM, which states that patients should receive support to self-manage their condition [17].

#### Goal Setting

Overall, 6 parents liked the idea of short-term goal setting within an intervention. Executive dysfunction theory has been applied to ADHD [60], and it states that CAYP with ADHD commonly experience working memory deficits. This is supportive of the fact that goals should be shorter rather than longer-term as the working memory capacities may not enable them to remember requirements to achieve a long-term goal.

**Figure 3:**
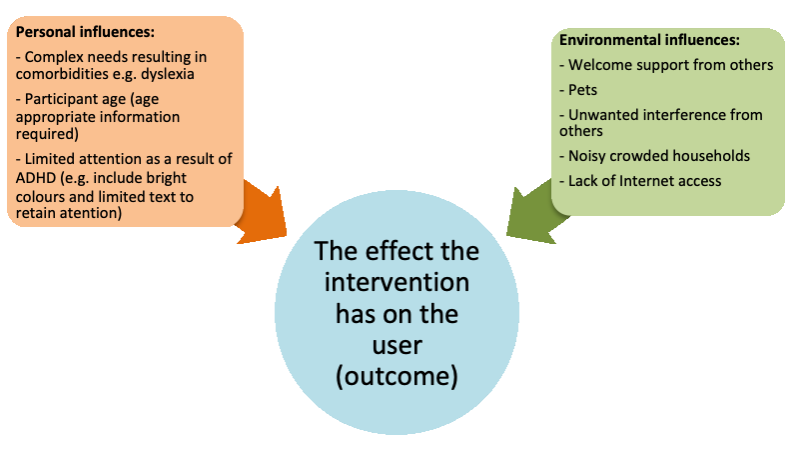
Outlines identified environmental and personal contextual factors that could affect the effect (outcome) an intervention has on a user.

#### Context: Environmental

In accordance with the MRC framework and the International Classification of Function, Disability and Health (ICF), this research found that variable contexts in which an intervention could be delivered could be divided into personal and environmental factors [79]. Figure 3 displays identified environmental and personal contexts as having the potential to change intervention outcomes.

Moreover, 1 example of an environmental context is support from others. A total of 13 participants believed there was value in CAYP with ADHD having support and encouragement (to use an intervention) from their close friends and families. This concurs with the theory of self-efficacy that states that “feedback and persuasion” from significant others, such as family members, can increase one’s self-efficacy [69,80]. Therefore, the support from a close friend or relative when completing such an intervention could help increase the user’s self-efficacy.

#### Context: Personal

Overall, 1 participant acknowledged that ADHD is a highlight comorbid disorder [81], which includes other conditions such as dyslexia, and 1 provision that could be made is giving users the option to change text background color to aid reading. In addition, optimal stimulation theory states that CAYP with ADHD need to be optimally stimulated to maintain their attention. Therefore, it is important that information and language presented are both age appropriate and interesting to look at, for example, by the use of bright colors.

### Strengths, Limitations, and Recommendations

This study has highlighted the importance of considering the variable context in which interventions take place [44]. If research does not consider factors such as the context the intervention is delivered in and the variety in the population, the results could lack reliability and depth [82]. Therefore, this study has provided initial guidelines to assist future technology developers with this process. Furthermore, MRTs were used to underpin the guidelines to help increase their generalizability to more than 1 context. Future research into complex intervention development for any population may wish to adapt the methodology of this study to assist with building an evidence base for the population’s intervention.

Existing evidence is supportive of a psychoeducation component for such interventions [49,73-76]; therefore, future technology should include this component if appropriate.

In addition, the BCW [21] provides a framework for behavior change interventions and the CCM [17] for the care of chronic conditions. During the production of these guidelines, the National Institute for Health and Care Excellence (NICE) released its digital health intervention (DHI) framework [83]. This is an excellent framework that makes a number of detailed recommendations for the development of complex DHIs. These models, and the NICE DHI framework, are valuable for behavior change, chronic care, and complex intervention development, respectively, they are generic models that can be applied to many conditions, not only ADHD. Where the guidelines developed in this instance are partially based upon generic theories such as these, they are also condition specific. This is important for a population with complex needs, such as CAYP with ADHD, as they have needs that cannot be applied to the many conditions the BCW, CCM, and NICE DHI framework target.

Although a sampling frame was adhered to, ensuring a representative sample of this complex population, the qualitative nature of this research meant that the CMOCs for this study were validated and refined using interview data from a small number of participants (n=21). ADHD is a highly complex neurological condition; therefore, 1 intervention will not suit all CAYP with ADHD, or all families and future technological interventions will need to account for this. In addition, for some, these guidelines and subsequent technology development may still not meet their need for personable one-to-one interaction. Furthermore, this study was limited to the views and opinions of CAYP with ADHD, their parents, and specialist clinicians. Game designers and platform developers were not consulted as it was outside the aims and objectives of this study. Future research may benefit from incorporating the views and opinions of these individuals.

Complex interventions for ADHD self-management run the inevitable risk of variable uptake of the intervention among participants [15]; therefore, future attempts should account for this. These guidelines were designed in 2018. Technology is constantly changing and alongside this, so are consumer expectations [43]; therefore, it is important for these guidelines to be reviewed regularly and for future projects to develop complex interventions to be aware of technological developments at the time. Although these guidelines may need reviewing, contexts that complex interventions are delivered in will always be variable; therefore, the methodology adopted for this study could be used beyond the lifetime of the guidelines developed.

### Conclusions

This study has adopted the principles of RE [52] to design a set of guidelines that can be used when developing complex, technological interventions that aim to help CAYP aged 8 to 11 years with ADHD self-manage their condition. The guidelines propose helping CAYP aged 8 to 11 years with ADHD understand their condition and providing them with tools to self-manage it more effectively. This concurs with the health foundation’s guide to self-management of long-term conditions [22]. It is anticipated that these guidelines will become a research derived actionable tool [84] in the future for designers to use and maximize the impact they have on the development of technological interventions for this population. It is recommended that a co-design approach should be adopted when designing complex interventions to increase the likelihood of acceptance of the intervention and engagement with the intervention [13,14]. The methodology presented could also be used to stimulate a wide range of stakeholders (service users, clinicians, researchers, and policy makers) to think differently about how interventions for this population, and other populations and age groups, are designed. Beyond the use of these guidelines, future research evaluating the effectiveness of such an intervention must contain large sample sizes and account for the variable contexts interventions are delivered in to ensure that the findings are generalizable. A follow-up period is also essential to evaluate if intervention effects persist over longer periods of time [15,49]. Although these guidelines provide a good theory and evidence basis for the development of a future complex intervention of this nature, it must be acknowledged that it is vital that complex interventions should be codesigned in partnership with key stakeholders to increase the likelihood that the intervention is to be accepted by the intended users [46-48].

## Appendix

Multimedia Appendix 1 The guidelines for the development of technological interventions for Children and young people with attention deficit hyperactivity disorder.

Multimedia Appendix 2 Refined context mechanism outcome configurations.

## Abbreviations

ADHD: attention deficit hyperactivity disorder
ASD: autism spectrum disorder
BCW: Behavior Change Wheel
CAYP: children and young people
CCM: Chronic Care Model
CMOC: context mechanism outcome configuration
COM-B: Capability Opportunity Motivation-Behavior
DHI: digital health intervention
MRC: Medical Research Council
MRTs: middle-range theories
NHS: National Health Service
NIHR: National Institute for Health Research
NICE: National Institute for Health and Care Excellence
OIT: Organismic Integration Theory
RCT: randomized controlled trial
RE: realist evaluation
