# Supplementary material for: Guideline Development for Technological Interventions for Children and Young People to Self-Manage Attention Deficit Hyperactivity Disorder: Realist Evaluation
Source: J Med Internet Res. 2019 Apr 3;21(4):e12831. doi: 10.2196/12831 (PMC6468334; doi:10.2196/12831)
Supplement: Multimedia Appendix 1 [file jmir_v21i4e12831_app1.pdf]

Appendices 1. An outline of eleven recommendations based on the refined CMOCs generated from this study. These recommendations comprise of a set of guidelines that are aimed at anybody who wishes to develop a technological intervention in the future to help CAYP with ADHD manage their condition. Alongside each recommendation is a set of circumstances or environments where the intervention is recommended to be delivered in.

	**Recommendation**	**Context the recommendation should be delivered in**
1	The user (CAYP with ADHD) should receive positive rewarding feedback (visual and audio) as it may improve their confidence by confirming performance.	There should be Internet access and the intervention should be accessible at home and used independently of clinician. The intervention should be colourful and not too text heavy.
2	Users could have the option of using downloadable gaming resources as this may mean the user can generate a deeper understanding of concepts covered in the intervention.<br>Examples of downloadable resources: Quizes, maze, word search, cross words, colouring in pictures, origami	The paper downloadable gaming resources should be available to be used independently or with support from close friend/relative by the user.
3	The intervention should enable the user to choose personalisable and adaptable characters of majority and minority groups. Additionally there should be a limited number of "modules" to help the user maintain stimulation to carry out the task.	The intervention should provide the user with lots of choice to keep them engaged and motivated. Users should also have their own user area so they can return to previous work and carry on where they left off.
4	Positive reinforcement should be incorporated into the intervention (personalised collectable rewards) as this may motivate the user to use the intervention.<br>Examples of rewards include: Diamonds, coins (use to buy items on game e.g. skins, avatar accessories), certificates, medals, personalised reward, tokens	The intervention should give persoanlised collectable positive and rewarding feedback to the user.
5	The intervention could include animated social scenarios, which may help the user make more appropriate social decisions, which may help enhance social relationships.<br>Another option would be to use animals instead of human characters/avatars.	The intervention should positively reward appropriate decision-making during animated social scenarios provided.
6	The intervention could help the user to have a better understanding of their ADHD.	The intervention should provide age appropriate information that should improve the user's knowledge and understanding of their ADHD.

7	The intervention could encourage the user to involve their parents/carers/close friends/family members at select times when they use the intervention. For example the user could teach their loved ones ways they have learned to help manage their ADHD. This is because the intervention could help to improve relationships and ADHD symptom self-management	The intervention should provide age appropriate information to improve the user's knowledge and understanding of their ADHD including strategies of how to calm them down when they feel angry.
8	The intervention should make it clear that encouragement from close friends and or relatives could reinforce the users' engagement with the intervention.	The intervention should be used in an environment where the user is encouraged by close friends and or relatives to engage with it.
9	The intervention could help the user to set short-term relevant meaningful goals for themselves which may encourage the user to engage with the intervention and self-manage their ADHD more effectively.	To incorporate achievable short-term goal setting for the user or to provide advice on how to set short term meaningful goals. Additionally, downloadable resources could incorporate encouragement for parents to think about short-term goals with their child.
10	The intervention could enable the user to have a better understanding of their ADHD so they can explain it to others (friends/family).	The intervention should provide age appropriate information to improve the user's knowledge and understanding of their ADHD and provide suggestions of how to explain their ADHD to others.
11	The intervention should provide an indication of improvement/progress during activities within the intervention such as leveling up will motivate adherence	The intervention should provide the user with varying game levels to keep them engaged and motivated.<br>A "simplify option" (to make the level more manageable for the user if they are struggling to complete it) could also be available to keep frustration levels down where applicable.
