# Supplementary material for: Guideline Development for Technological Interventions for Children and Young People to Self-Manage Attention Deficit Hyperactivity Disorder: Realist Evaluation
Source: J Med Internet Res. 2019 Apr 3;21(4):e12831. doi: 10.2196/12831 (PMC6468334; doi:10.2196/12831)
Supplement: Multimedia Appendix 2 [file jmir_v21i4e12831_app2.pdf]

Appendices 2. Presenting the CMOCs that have been refined (CMOCs1-9) and developed (CMOCs 10-11) as a result of this study. Bolded text shows where refinements have been made to the original CMOCs and also where new CMOCs have been developed.

**CMOC's**	**Plausible mechanism "What"**	**Contexts: "for whom" and "in what circumstances"**	**Possible outcomes**
CMOC1	Receiving **positive rewarding** feedback from ADHD Hub^{TM} might improve the users confidence by confirming performance.<br><br>**Include audio feedback**	Internet and intervention should be accessible at home and used independently of clinician. **Intervention should be colourful and not too text heavy.**	Development of self-efficacy
CMOC2	Having the option of using downloadable **gaming** resources may mean the user can generate a deeper understanding of concepts covered within the intervention<br>**Downloadable resources options: Quizes, mazes, word searches, cross words, colouring in pictures, origami.**	Paper downloadable **gaming** resources available to be used independently **or with support from close friend/relative** by the young person with ADHD.	Improved performance on exercises provided by the intervention.
CMOC3	Enabling the user to choose **personalisable and adaptable** characters **of majority and minority groups** and **a limited number of** "modules" will maintain stimulation to carry out the task.	The intervention will provide the user with lots of choice to keep them engaged and motivated to use theintervention. **Users will also have their own user area so they can return to previous work and carry on where they left off.**	Improved performance on exercises provided by the intervention.
CMOC4	Positive reinforcement (**personalised collectable** rewards) may motivate the user to use the intervention.<br>**Reward options: Diamonds, coins (use to buy things on game e.g. skins, character accessories), certificates, medals, collect trophy fragments to get trophy at the end, personalised reward, tokens**	The intervention will give **persoanlised collectable** positive and rewarding feedback to the user.	Increased understanding of condition and self-management.
CMOC5	**Animated** social scenarios provided may help the user make more appropriate social decisions, which may help enhance social relationships.<br>**Could use animals instead of characters/avatars.**	The intervention will positively reward appropriate decision-making during **animated** social scenarios provided.	Increased understanding of social situations.

CMOC6	User will have a better understanding of their ADHD	The intervention will provide age appropriate information to improve the user's knowledge and understanding of their ADHD	Increased knowledge and understanding of their ADHD
CMOC7	Use of the intervention could improve relationships and ADHD symptom self-management	The intervention will provide age appropriate information to improve the user's knowledge and understanding of their ADHD **including strategies of how to calm them down when they feel angry**	Improved quality of life, **improved self-management of ADHD.**
CMOC8	Encouragement from **close** friends and or relatives could reinforce the users' engagement with the intervention.	The intervention will be used in an environment where the user is encouraged by **close** friends and or relatives to engage with it.	Increased use of the intervention.
CMOC9	Setting short-term relevant meaningful goals may encourage the user to engage with the intervention.	The intervention will incorporate achievable **short-term** goal setting for the user.<br><br>**Or downloadable resources could incorporate encouragement for parents to think about ST goals with their child.**	Increased use of the intervention and behavior change (ADHD self management).
**CMOC10 (New CMOC)**	**User will have a better understanding of their ADHD so they can explain it to others (friends/family)**	**The intervention will provide age appropriate information to improve the user's knowledge and understanding of their ADHD and provide suggestions of how to explain their ADHD to others.**	**Increased knowledge and understanding of their ADHD**
**CMOC11 (New CMOC)**	**An indication of improvement/progress such as leveling up will motivate adherence**	**The intervention will provide the user with varying game levels to keep them engaged and motivated to use the intervention.**<br>**A "simplify option" could also be available to keep frustration levels down where applicable.**	**Improved performance on exercises provided by the intervention.**
